# Observations on Yellow Fever and Its Treatment

**Published:** 1881-07

**Authors:** Harvey L. Byrd

**Affiliations:** Formerly Professor in the Savannah Medical College, and Oglethorpe Medical College, of Georgia; late Professor in Washington University, of Maryland, etc., Baltimore; 225 N. Gilmor Street, Baltimore


### Independent Practitioner.

VOL. II.——*July*, 1881.——NO. 7.

ARTICLE I.

### OBSERVATIONS ON YELLOW FEVER, AND ITS TREATMENT.

BY HARVEY L. BYRD, A. M., M. D.

Formerly Professor in the Savannah Medical College, and Oglethorpe Medical College, of Georgia; late Professor in Washington University, of Maryland, etc., Baltimore.

In view of the widespread interest existing throughout the country, in and out of the profession, resulting from the devastating epidemics of yellow fever that have frequently scourged and desolated so many cities and towns in our Southern States, and to "vindicate the truth of history," the following paper has been prepared. The subject is most deeply interesting and important to us all, whether we consider the circumstances connected with its introduction and spread in our country, the pecularity of its visitation, the extent and terror of its ravages, or the loss of prosperity and wealth to the cities the unfortunate victims of its visitation. Whether, indeed, it is regarded from a hygienic, etiological, pathological or therapeutical standpoint, it is of the profoundest interest, and certainly demands the most earnest and careful thought and investigation of every physician who really desires the advancement of his profession, or regards the relief of human suffering and preservation of human life, objects of great importance.

It is to be regretted that conflicting, and, in many instances, directly contradictory statements are on record from medical men, even up to a recent date, whose opinions, we would be led to suppose, from their surroundings and opportunities for the study and investigation of the disease, ought to entitle their utterances to credence and respect. But interest, prejudice, or disposition to bend and accommodate ascertained facts to commercial wants and requirements, have all tended to render confusion worse confounded on almost all questions growing out of yellow fever investigations. From the days of the immortal Rush, the tendency of commercial men has been to repudiate medical opinions adverse to, or in conflict with, their interests and views of the epidemical visitations of yellow fever in their communities. But, thanks to the more careful study of zealous men, an earnest desire for truth, whatever it might be, has induced them to place on record their honest and candid convictions, after thorough investigation of the disease. And, therefore, much of its true nature and characteristics, as thus developed and understood, are now before the profession.

For more than a quarter of a century, yellow fever has been a subject of the deepest interest to myself; and during a decade of that time, it claimed as close attention and study as I have ever given to any matter whatsoever. At the bedside, on the cadaver, and from books, information was zealously sought, and the revelations of Nature carefully systematized and compared with the observations and statements of others. The consequence was that more satisfactory and correct views were reached in regard to its natural history, transmissibility, contagiousness, etc.; and it is gratifying in the highest degree to find those opinions and views have also been reached by others in different, and in one instance, at least, remote fields of observation and experience; and that, too, by gentlemen of high and enlightened positions in the medical profession. In making these statements, no arrogation of superior intelligence or greater industry than many others who have labored in the same field, is attempted; but the ground was most inviting, and the chances for a large yield of valuable fruit so promising of reward for the labor bestowed on its culture, that the work partook of pleasure and enjoyment in all its details, and hence the results.

Thirty years ago, yellow fever was regarded by some prominent practitioners, even in our Southern cities, as an aggravated or malignant type of bilious remittent fever, with increase of some of its graver symptoms. When inquiry was made in regard to its very rapid course and peculiar mode of termination, the answer was that the type of the remittent was more *congestive* than usual.

In an article on yellow fever, published in the *Charleston Medical Journal and Review*, volume X, page 329, May, 1855 (the year succeeding the terrible epidemic in Savannah, 1854) I used the following language : "I know little of the opinions of my contemporaries, but I have formed my own. I believe, with a condition of the atmosphere *favorable* to its extension, it is as much a *contagious* disease as measles or small-pox." As proof the *contagiousness* of the disease, the "parallelism" of exemption from *second* attacks of *other* contagious diseases was introduced ; and in the same article, at page 333, occurs this language :

"From all the data I have been able to procure, this exemption or protection against a second attack is *equal* to that afforded by an attack of measles or small-pox against subsequent ones. Second attacks of these two last named diseases do occasionally occur, but the entire profession is aware they are exceptions to a rule. The *fact* that one attack affords protection against a subsequent one of yellow fever, is so well known and understood by the inhabitants generally, as well as by the physicians of our Southern cities, it would be supererogatory work to add much more in regard to it. Does not this *fact* (for fact it is) add additional weight to the statement of the contagious or transmissible character of yellow fever ?"

In regard to the distinct character of the disease, it is stated at page 329 (same article) that—

"Yellow fever is a disease separate and distinct from the remittent form of fever which annually visits our Southern country, including the cities and towns, and it bears no more affinity to continued typhus and typhoid fevers than it does to remittent or bilious fever, as it is called." "It is now regarded by the profession generally, that *malaria* is the source of all the forms of intermittent and remittent fevers we encounter in the Southern and Southwestern States. If this proposition be true, is it certain, or even probable that the poison which produces an intermittent will produce typhus or typhoid fever ? No one in the legitimate profession, at this time, entertains an idea that measles and small-pox result from the same virus, or that syphilis and gonorrhœa are one and the same disease. I apprehend that the laws which govern the physical world generally are not more unvarying in their operations than those which govern the system in a state of health, and influence it in the development of disease."

In the same article, a number of instances are mentioned in which the contagious character was unmistakable, and as clearly established as anything could be by induction, or recognized facts, and to which the reader is respectfully referred, should he desire further evidence upon that point. And it is finally shown, in a conclusive manner, I think, that *quarantine* affords the *only* sure prevention against the transmissibility and propagation of yellow fever.

The subject of the *importability* and *contagiousness* of yellow fever is referred to again in an article in the *Oglethorpe Med. & Surg. Journal*, volume I, page 287 (1858), written and published during the existence of the epidemic of yellow fever in Savannah in that year, in which I used the following language: "I have every reason for believing the yellow fever epidemic of this year (1858), was imported into Savannah by the U. S. Mail Steamship 'Catawba,' of the Charleston, Savannah and Havana line, which received some repairs in the dry dock in the river, opposite the city, the latter part of July, and coaled at a wharf of the city, on the 30th of the month, ere her departure for Charleston." She came direct from Havana (where the yellow fever was prevailing) to Savannah, for some slight repairs to her machinery. The steamer had sickness on board whilst at Savannah; and on August 28th, 1858, a letter was received from Prof. Wm. Hume, M.D., of Charleston, stating, "The day after her arrival (Catawba) in Charleston, she sent one of her men to the Lazaretto, and it is to her men, who came up to the city, we owe the present (1858) epidemic." (See same Journal, vol. I, page 292).

In the same article, page 300, occurs the following remarks:

"From careful analysis of all the information I have thus far been able to procure, I am led to the following conclusion in regard to yellow fever, viz.: It is a disease *sui generis*—peculiar—bearing no closer relation to any other form or variety of fever than the affinity that may be said to exist between measles and small-pox, or that of any of the other exanthematous diseases, as dissimilar in their *essential* nature and characteristics; that it is capable of being transported beyond seas, etc." "As the foregoing conclusions admit of demonstration, it behooves us, citizens of Savannah, to consider well the following propositions, viz.: 1st. The propriety of prohibiting *all* commercial intercourse between Savannah and the West Indies, or other infected ports, *for the period of the year during which the temperature of our climate approximates that of those Islands*, and therefore favors the spread of the yellow fever germs, when once introduced amongst us ; and 2d, Whether the *actual* and *prospective* losses sustained by the city, in money, during each epidemical visitation of yellow fever, are not far beyond *all* the wealth the commerce of the Indies brings to our seaport in a decade of years. I have said nothing of the suffering and loss of life, as neither of them can be weighed or estimated by *any* standard of money valuation."

I had the honor of reading a paper before the Epidemiological Association of Baltimore, when President of that body in 1873, on subject of yellow fever, and I beg to present a brief *resumé* of the leading facts and conclusions reached in that paper, as published in the *Philadelphia Medical Times*, volume III, page 726. Before doing so, however, I may, I trust, premise, without appearing egotistical, that the facts therein contained have been fully sustained in *every* particular, by an able article in *Ziemssen's Cyclopædia of the Practice of Medicine*, to which attention will be called at a later period in this paper ; and also most fully repeated and sustained in the reports of Prof. S. Choppin, M.D., of New Orleans, and others, in the *Virginia Medical Monthly*, December, 1878. (*Vide* proceedings of the Public Health Association in Richmond, Va.) I said of the history of yellow fever in the *Philadelphia Medical Times*, August, 16, 1873 :

"Nothing concerning the source or origin of yellow fever can be obtained from the various names by which it has been recognized or described. They relate either to the place of visitation or invasion, or to some real or fancied pathological change of most frequent occurrence during the prevalence of the disease, in an epidemic form. It was probably first introduced into Southern Europe, the West India Islands, and the continent of North and South America from the *west coast of Africa, its natal place.* Had European ambition for extended dominion and territory, or the avarice and cupidity of the New England slave trader, never attempted the invasion of the jungles of Western Africa, there is reason to believe that the hecatombs which have marked the visitations of the pestilence in the West Indies and on the shores of this continent, at least, would never have been known. The autochthones, or aborigines of those lands, knew nothing of the disease until brought to them by ships engaged in a most unholy commerce. As it is chiefly with the disease as it concerns us of the United States we propose to deal, it may be observed that the summer, or warm season of the year, is the period during which its visitations have prevailed epidemically, and been most destructive in their character.

"*Locality.*—As it is comparatively unknown in the country, and confined almost exclusively to cities and large towns, as an epidemic, and as in those cities and towns holding commercial relations with tropical countries it always establishes itself before interior localities become affected, the *importance of strict quarantine regulations in such commercial cities, during the hot season,* cannot be over-estimated.

"It might prove interesting, did time permit, to introduce the date of its visitation to the several cities of our country, and the duration and severity of the same; but we must pass on, merely mentioning a few of the principal cities in which it has prevailed, viz.: Boston, New York, Philadelphia, Baltimore, Norfolk, Wilmington, Charleston, Savannah, Mobile, New Orleans and Galveston. It will be observed that all the places named are seaport cities, or cities engaged in foreign commerce. From Charleston and Savannah it has been carried, when epidemic, to Beaufort and Blackville, S. C., and to Augusta and Darien, Ga.; from Mobile to Selma and Montgomery, Ala.; and from New Orleans to Natchez, Vicksburg and Memphis, on the Mississippi river, and other places of note, thus *proving that it is a transmissible disease.*

"*Season.*—With a protracted temperature, in our seaboard cities, of from 85° to 95° F., the *introduction of the yellow fever germ, or fungus, will as certainly develop that disease in the unacclimated Caucasian,* as the introduction of the small-pox germ will give rise to that scourge in an unvaccinated community.

"*Cause.*—From what has just been stated, it will be seen that we believe the disease to depend upon a *specific cause,* and that cause to consist of *organic matter.* This theory is sustained by a number of well-attested facts. Like much of the flora indigenous to hot climates, the yellow fever germ, or fungus, requires for its proper development and growth a certain and continuous temperature.

Eighty-five to ninety or ninety-five degrees of Fahrenheit is the temperature which experience has shown to be most favorable to the spread of yellow fever epidemically in this country; and like exotic plants, when *exposed, the yellow fever germ is effectually destroyed by frost.* In the West Indies, and in the tropical districts of this continent, where the summer temperature is perpetual, these germs have become naturalized, so to speak, and the disease is there found to be perennial [in some of the cities located in this zone] by the continuous or frequent introduction of the unacclimated. The winters in all the sea-coast cities in this country, except, perhaps, New Orleans and Galveston, are too cold for yellow fever germs to be perpetuated; and they must, therefore, be reintroduced from season to season, in order to the production or development of the disease. It is a disease *sui generis*, as contagious, under favorable circumstances, as small-pox, measles or whooping-cough. Assertions of this character are not made lightly, or without due consideration of their import and value. *It bears no striking resemblance to any other form of disease*, and when once carefully studied at the bed-side, and in the dead-house, can *never be mistaken* by an intelligent observer *for any other affection or disease.* When *the important test* is applied that is recognized as of such *uniform value in other forms of contagious disease*, it will be found to respond with *much greater* emphasis and distinctness in yellow fever *than in any other disease*, viz.: *One well-defined attack of yellow fever protects the survivor with almost absolute certainty* from a subsequent one.

"*Race.*—The *native* African is *exempt* from the disease as it has prevailed in this country, and even his descendants for four or five generations, in our Southern cities, enjoy great immunity from it. Dr. W. C. Daniel, a former distinguished practitioner in Savannah, states that in the year 1820, when yellow fever devasted that city, 'nearly 300 native Africans, recently captured on the coast by government vessels, were brought to Savannah, and remained there during the epidemic, and *not one of them took yellow fever.*" The writer has never seen a case of *black vomit* in a genuine negro, however long his ancestors may have resided in the South. The most susceptible race is the Caucasian, and the purer the type the greater the liability to the contagion. The mulatto occupies, so far as susceptibility to the fever is concerned, an intermediate place between the Caucasian and

African races—being less liable than the former, and more exposed to an attack than the latter—his liability or exemption being dependent on the greater or less amount of Caucasian blood, and so on—in the further admixture of the above races—the susceptibility or liability to the fever being in exact ratio to the amount of white blood in the individual.

"*Miasma.*—Marsh effluvium, the prolific source of intermittent and remittent or bilious fever, so-called, *is not* the source or cause of yellow fever. In certain districts and communities, where bilious fever is an annual visitant, and where its appearance is looked forward to with the greatest dread and apprehension, yellow fever is totally unknown. Take Georgetown, S. C., for example, which is situated in the midst of the rice-growing region of the Pee Dee and Waccamaw Rivers. It has never suffered from a visitation of yellow fever, though on the coast, and only about fifty miles from Charleston. Yet in this notoriously insalubrious climate—though a bilious fever has scourged it more or less frequently since its early settlement, and with a violence, it was said, a few years ago, unsurpassed by any other town in the world, except Alexandria, in Egypt—a case of yellow fever has never originated within its limits. Yellow and bilious fevers may exist or run *pari passu* for a time in certain localities; and from this circumstance, doubtless, the former idea of its *miasmatic origin* was conceived. This hypothesis is erroneous, however, as the two diseases differ as widely in their symptoms and pathological appearances as any two other known diseases whatsoever.

"*Contagion.*—In the sense in which the term is usually understood, yellow fever is a *contagious disease.* Not only have the germs of yellow fever been carried from an infected to a healthy community by individuals, but even the clothes of persons on dying from the disease, have conveyed the germs, and infected, when exposed and handled, relations and friends in healthy communities hundreds of miles away. You may denominate the contagion of yellow fever a *contingent* one; but it is, nevertheless, a contagion."

The value of this paper will be further enhanced by the introduction of the observations and experience of others, whose opportunities for investigation and study of yellow fever give *their corroboration* and endorsement of the *principles and facts* it represents—a value intrinsically more important, if possible, than their historical and chronological preservation in the literature of our science. In *Ziemssen's Cyclopædia*, volume I, page 480 (October, 1874), after speaking of the theories in vogue in regard to the causes necessary to the development of an epidemic of yellow fever, are these remarks, viz.:

"To bring about an epidemic of yellow fever anywhere, a series of conditions must be fulfilled, which are deducible from a comparison of the history of single epidemics. These conditions are allied partly to the external circumstances of human beings; partly to those which are personal. Among the external circumstances are climate and terrestial conditions, which must present a certain definite character. The mean temperature of the year must reach at least 72 deg. or 77 deg. Fahr.—such a temperature, in fact, as is always observed in the tropics, especially in the Antilles. It is, indeed, maintained by Griesinger, that the temperature *must be uniformly hot* for a considerable time (80 deg. Fahr)."

At page 490, it is stated, "Touching the terrestial circumstances, it is particularly noticeable that the disease is chiefly developed in cities, and, moreover, in cities which have maritime commerce, whether they be upon the sea-coast or upon important rivers. Here it is almost invariably in that quarter of the city lying nearest to the harbor which is the first to be attacked in every new epidemic, etc."

After speaking of malarial influences on land, and sea-going ships, the writer remarks (page 491):

"Still, though *all* the unfavorable conditions mentioned may have been developed on board in the *highest degree*, *the yellow fever has never yet been observed on a ship* which has not in some way *come into communication with the land*, or with *some other ship*, where the disease *already prevailed*."

On the same page, the writer says:

"As regards the influence of *race*, the history of every separate epidemic has established the fact that the *negro* race possesess an *almost* absolute immunity. Only such negroes were known to contract the disease as had lived for a considerable time in the temperate zone, thereby forfeiting their immunity, and then had returned into the yellow fever district shortly before an outbreak of the epidemic. Moreover, fatal epidemics *have been observed among the blacks on the west coast of Africa, where yellow fever prevails endemically.*"

A full endorsement is thus given to my theory of the source from whence our calamitous epidemics *originally* sprang. On page 492, when speaking further on the subject of *race*, the writer says :

"The whites are *most exposed* to danger. Between the two, stand the *mixed races*, and in fact, those are just so much more subject to the infection the more *white blood* flows in their veins, and the paler their skins are." "This susceptibility, however, gradually diminishes the longer these whites have lived in the tropics, in the regions where the yellow fever is endemic ; it is almost entirely extinguished if they have passed through an epidemic, even without themselves being sick, and it is finally *absolutely extinguished after once experiencing the disease.*"

As to the cause of the disease, it is stated on page 493 of the same article :

"Yellow fever is most probably produced by a *living* miasm, which has hitherto entirely eluded microscopic demonstration, but the existence of which is argued from very many facts. These seeds of disease, as soon as they become in any way established in a human organism, set up in it that diseased process to which we are accustomed to give the name "yellow fever."

From the foregoing extracts, from Ziemssen, the reader is doubtless prepared to learn the following in regard to prevention of the disease. At page 510, of the same article, we read :

"The government ordinances must extend to a strict police supervision of the houses, streets and harbor, in places where the disease is endemic, and in other districts they must seek to prevent the importation of the poison, by the enforcement of quarantine regulations. It is not possible absolutely to prevent an introduction of the yellow fever poison, even by a strict quarantine. To accomplish this, the same judicious laws would have to be in force in all ports ; and, even then it would be possible to establish communication between an infected ship and the shore by means of boats at unguarded points; and then there is *always a possibility* of conveying poison by land from an infected port to one hitherto free, by means of goods sent by rail, or by means of the personal effects of the men. But this would, in any event, be only exceptional, and there need be no question that important protection is to be gained by wise quarantine."

On page 511, same article, as the caption of a most emphatic and important paragraph on *quarantine*, occurs this sentence, viz :

"Every ship *must be subject to quarantine*, which has communicated with an infected port, or an infected ship, even if no case of disease has occurred on a voyage of some weeks' duration. That is to say, the human beings may possess no susceptibility to the yellow fever, may even remain healthy, and yet the poison of the disease conveyed in clothing, personal effects, cargo, or bilge-water, may have retained its capacity for infection."

The value of the foregoing extracts from *Ziemssen's Cyclopædia* will be enhanced in the estimation of some readers, when informed that the author of the article on yellow fever (Dr. Fritz Haenisch), from which these excerpts are made, was a medical officer in the German Navy. As such, he had the opportunity of visiting the Mediterranean, Madeira, the West Indies, South and North America, the Azores and Portugal. (See *Ziemssen's Cyclopædia*, volume I, page vii.)

With a few gleanings from the December (1878,) number of the *Virginia Medical Monthly*, in its report of the "American Public Health Association," this paper will be brought to a close. Prof. S. Choppin, a native of New Orleans, and President of the Board of Health of that city, is reported (at page 764) as saying, in his paper before the Association, that the yellow fever of 1878 was "brought from Havana to New Orleans, by the steamship Emily B. Souder.

"The experience of the present year, (1878,) with regard to the efficacy of a *strict quarantine*, goes to sustain the theory of importation and portability of yellow fever. Witness Galveston, which has not developed a single case ; witness Shreveport, Monroe, La., and Natchez, Miss., with their shot-gun quarantines turning away pestilence ; and witness, again, Mobile, which has certainly escaped an epidemic." "From the statement and facts given in the accompanying papers, it is an irresistible conclusion, that yellow fever is *not* an indigenous disease of Louisiana, or any other part of the United States ; but that the many years when it has made its appearance in this country, it could be traced either directly or remotely to foreign source." "The great object to be aimed at is to prevent the germ or fomites of this dreaded pestilence from having access to our people; and the only certain and sure prevention of yellow fever, in my humble opinion, is *absolute non-intercourse* with ports where yellow fever is indigenous from the first of April to the first of November of each year." "Once eradicate this disease from the land, as it must necessarily be by our cold winters, and, mark my word, we will never again be visited by the terrible scourge unless introduced from abroad."

Dr. Choppin estimates the cost of that yellow fever epidemic to New Orleans at over $12,000,000, which we have no doubt is rather under than over the true amount.

Dr. W. G. Austin, also, member of the New Orleans Board of Health, and who has been familiar with epidemics of yellow fever in that city since 1839, after comparing the epidemic of that year with the one then just past, says:

"In thirty-five years' experience in Louisiana and Mississippi, *he was satisfied* there has never been a single case originated there." "It is brought to us in ships in fomites." (*vide* page 765.) "It is *not* endemic in any of the Southern States." "Dr. Austin proposes that 1878 shall be the last year of yellow fever in New Orleans, if we shall declare *non-intercourse* between all the ports where yellow fever is endemic, and New Orleans, from the first day of May, until the first of November." (*Vide* page 766.)

The conclusions reached by the Commission appointed by the head of the Marine Hospital Department, to examine into and report on the epidemic of 1878, as it prevailed in the Mississippi Valley, are not less emphatic in their utterance in regard to the importation of the disease, and its communicability or contagious character. When it is remembered that the first two gentlemen on the Commission have had many years' experience in the management of yellow fever, it will be seen that the evidence is strengthened in regard to importability. The last gentleman, though unacquainted with the disease practically, is, nevertheless, an intelligent observer of facts, and he joins fully with his colleagues in the following report, after some weeks residence in the yellow fever cities. At page 758 occurs the following, viz:

1. "We have not in a solitary instance found a case of yellow fever which we could justifiably consider of *de novo* origin, or indigenous to its locality. 2. In respect to most of the various towns which we visited, and which were points of epidemic prevalence, the *testimony showing its importation was direct and convincing in its character.* 3. The transmission of yellow fever between points separated by any considerable distance appeared to be wholly due to human intercourse. In some instances, the poison was carried in the clothing, or about the persons of people going from the infected districts. In other instances, it was conveyed in such fomites as cotton-bagging, or goods of some description, or bedding and blankets, etc. * * * * 6. Quarantine established with such a degree of surveillance and rigor that absolute non-intercourse is the result, has *effectually*, and without exception, protected those quarantines from attacks of yellow fever." "Each one of us has exercised the utmost care possible to be observed, that whatever facts we might gather and lay before you *should be facts in reality*." Thus much for the labors of the Commission.

It is stated on page 760, "*Mulattoes suffered* more from fever than *full-blooded negroes*." This is an important fact,and,though briefly stated, conveys a most valuable item of information to the ethnologist, as well as physician, and is in full accord with my own and the observation of all practitioners of much experience in the treatment of yellow fever.

The "importation theory" of the disease received the endorsement of many able and distinguished gentleman of the Association, such as Dr. S. S. Herrick, of New Orleans, Dr. Sternberg, of the U. S. Army, Dr. Mitchell, of Memphis, Dr. Guihon, U. S. Navy, Dr. Trescott, of Charleston, and Dr. Wm. Seldon, of Norfolk.

From the experience and testimony adduced in this article, it will readily be seen that a greater degree of unanimity exists among leading and educated physicians, in regard to the nature and prophylaxis of yellow fever than was ever known before. And it is earnestly hoped the revelation of science may *compel* commerce to yield to the demands of humanity, in affording effective quarantine against yellow fever in the future. The foregoing was chiefly published in the *Virginia Medical Monthly*, but having been urged to give my opinions in regard to the *importability* and the *treatment* of yellow fever, it is reproduced, as my opinions remain unchanged since its publication, and it properly introduces what may be said upon the *treatment*.

### TREATMENT.

We have always endeavored in the treatment of diseases to be guided by as intelligent and rational views as we were capable of bringing to bear in each individual case, taking into account the mental and physical state or condition of the patient and his surroundings, including climate, season of the year, etc., and a case of yellow fever, sporadic or epidemic, has not proven sufficiently grave in its import and characteristics to induce a departure from this rule of action. In what we may say about the treatment of yellow fever, we wish our readers to bear in mind, *ab initio*, that climate and season of the year, and their impression and influence upon the sick, must not be lost sight of in estimating its value and importance for a single moment.

The mental state or condition of the patient should next claim the prompt and earnest attention and consideration of the practitioner. And we should, perhaps, as well remark here as elsewhere, that the physician who is successful in *calming* the fears and *inspiring* hope in the mind of his patient will soon learn, from experience, that he has taken a most important step in controlling the case in doing so—quite as much so, in fact, as could be effected by any other agent at his command in the treatment of an adult case of yellow fever.

The best method of gaining this important mental influence and control must depend necessarily upon two factors, viz: the physician and the patient himself. The proper relationship having been established between them, it is easy enough to calm the fears and perturbations of the latter to such an extent as to render him amenable to further treatment. The next step is to enjoin, and if practicable and necessary, enforce *the recumbent position undeviatingly*, until convalescence ensues, or the crisis of the disease has been safely passed. By adopting this course and attending carefully to the therapeutical indications to be spoken of by and by, the mortality in yellow fever should not exceed 5 per cent. Indeed, in our own experience, it has never marked 5 per cent., and in one epidemic it was not quite 4 per cent.

The air in the room of the yellow fever patient should be kept fresh and pure, and the apartment rendered as cool and comfortable as practicable, and pounded ice, or small portions of ice-water, given *pro re nata.*

THERAPEUTICS.—When the cerebral symptoms are strong and attended with coma, unconsciousness, or raving delirium in the inception of the disease, with strong and tense pulse, we should *bleed*, just as should be done in pneumonia, or any other disease calling as loudly for the use of the lancet. Sometimes leeches and cups can, and should be substituted for general blood-letting. But we assert, without the slightest fear of satisfactory refutation, that we have no means at our command that ought to be substituted for sanguinous depletion in the condition just named ! And all who may try it once under such circumstances will never fail to repeat it, again and again, in the management of those symptoms ! Ice is also valuable, applied to the head in this condition. We will observe, just here, that the treatment to be most successful in yellow fever is that which is prompt in application and efficient in action ; for it should ever be borne in mind that whatever is done for the relief of the patient must be done within a few hours !

Having cared for the cerebral symptoms, as above, *calomel*, in 5 to 10 grain doses, with 10 grains of pulv. *charcoal* and 5 grains of pulv. *extract of colocynth camp.* and ¼ grain of *pulv. capsicum*, should be given every two hours ; and as soon as the slightest moisture, or perspiration is observed upon the surface, 5 grains of *quinia*, and 1-12th grain *strychnia*, with 5 grains *pulv liquorice*, every two hours. Under this treatment the skin will usually grow more moist, and its heat subside. Should the bowels not move in six to eight hours, or should they become distended, or uncomfortable, before the action of the calomel combination, open them by pumping into them, in a gradual and gentle manner, from one to two pints of *tepid water*, into which one or two ounces of *common salt* has been dissolved. A supository of *opium* (2 to 4 grains) will give much comfort, as well as prevent too free action upon the bowels when they have been sufficiently moved. All nauseous and irritating cathartics, as castor oil, etc., should be carefully avoided. Should nausea or vomiting interfere with the treatment just detailed, or prove very distressing, the application of *chloroform* or *ammonia*, sprinkled over a piece of cotton batting, or a folded cloth five or six inches in diameter,shoud be applied to the epigastrium, and evaporation prevented by a piece of oiled silk, or oiled cloth of any kind, or even a towel folded and placed over the application, until the skin is thoroughly reddened. I prefer the above to the use of the *fly blister*, which may be used, however, should the nausea or vomiting persist. I have usually found that when a dose was vomited, another given immediately after the stomach was emptied in that way, would be retained. The foregoing constitutes the active treatment I have usually pursued in the first stage of yellow fever : To procure rest, and to relieve the usually distressing supra-orbital pain, a *hypoderm* of *morphia*, or *morphia* and *atropia*, in the usual dose, would quickly afford the needed relief. In the *second* stage of the disease, cold, or ice to the head gives great comfort, and often, after a hypoderm of morphia, affords complete relief. In the *third* stage, and sometimes earlier, stimulants of various sorts, particularly *champagne*, will be found of great value, and the *mur. tinct. iron*, with other tonics, should be used as required. Want of space prevents the mention of many remedies of greater or less value and importance in the treatment of yellow fever ; and we must bring our remarks to a close after a few words more.

We have tried almost all the modes of treating yellow fever now in vogue, and that have been heretofore recommended, except the irrational and inhumane one of "*sweating out the disease*," and though many years have passed since our first experience with yellow fever, we have had no regrets at not having *murdered* a poor yellow fever sufferer with a surrounding temperature of 88 to 95 deg. F., and a feeling of burning up with the fever, by putting him through "the sweating treatment." We honestly believe that a perfectly well, but *unacclimated* person, making his appearance in a southern city during the prevalence of an epidemic of yellow fever, and being told he had that fever, and then subjected to the "sweating treatment," would not stand one chance in ten for his life. In other words, there would be nine chances to one that he would not survive the treatment.

Only the salient points in the treatment of yellow fever are introduced in this brief article. But we hope to dwell somewhat at length upon it hereafter. What we consider to be the more rational and reliable means have been mentioned here.

225 *N. Gilmor Street*, *Baltimore*, June, 1881.

